# Supplementary material for: Antiferroelectric Order in Nematic Liquids: Flexoelectricity Versus Electrostatics
Source: Adv Sci (Weinh). 2025 Jan 9;12(9):2414818. doi: 10.1002/advs.202414818 (PMC11884549; doi:10.1002/advs.202414818)
Supplement: Supplementary file 1 — Supporting Information [file ADVS-12-2414818-s001.pdf]

ADVANCED SCIENCE
Open Access

Supporting Information

for *Adv. Sci.*, DOI 10.1002/advs.202414818

Antiferroelectric Order in Nematic Liquids: Flexoelectricity Versus Electrostatics

*Peter Medle Rupnik, Ema Hanžel, Matija Lovšin, Natan Osterman, Calum Jordan Gibb, Richard J. Mandle, Nerea Sebastián and Alenka Mertelj**

Supporting Information

Antiferroelectric order in nematic liquids: Flexoelectricity vs electrostatics

Peter Medle Rupnik[1,2], Ema Hanžel[1,2], Matija Lovšin[1,2], Natan Osterman[1,2], Calum J. Gibb[3,4], Richard J. Mandle[3,4], Nerea Sebastián[1], and Alenka Mertelj[1]

1 Jožef Stefan Institute, Ljubljana, Slovenia
2 University of Ljubljana, Faculty of Mathematics and Physics, Ljubljana, Slovenia
3 School of Chemistry, University of Leeds, Leeds, UK
4 School of Physics and Astronomy, University of Leeds, Leeds, UK

Corresponding author: alenka.mertelj@ijs.si

Contents

Pages 2-10 Supplementary Figures S1 to S11

Pages 11-13 Supplementary Note 1

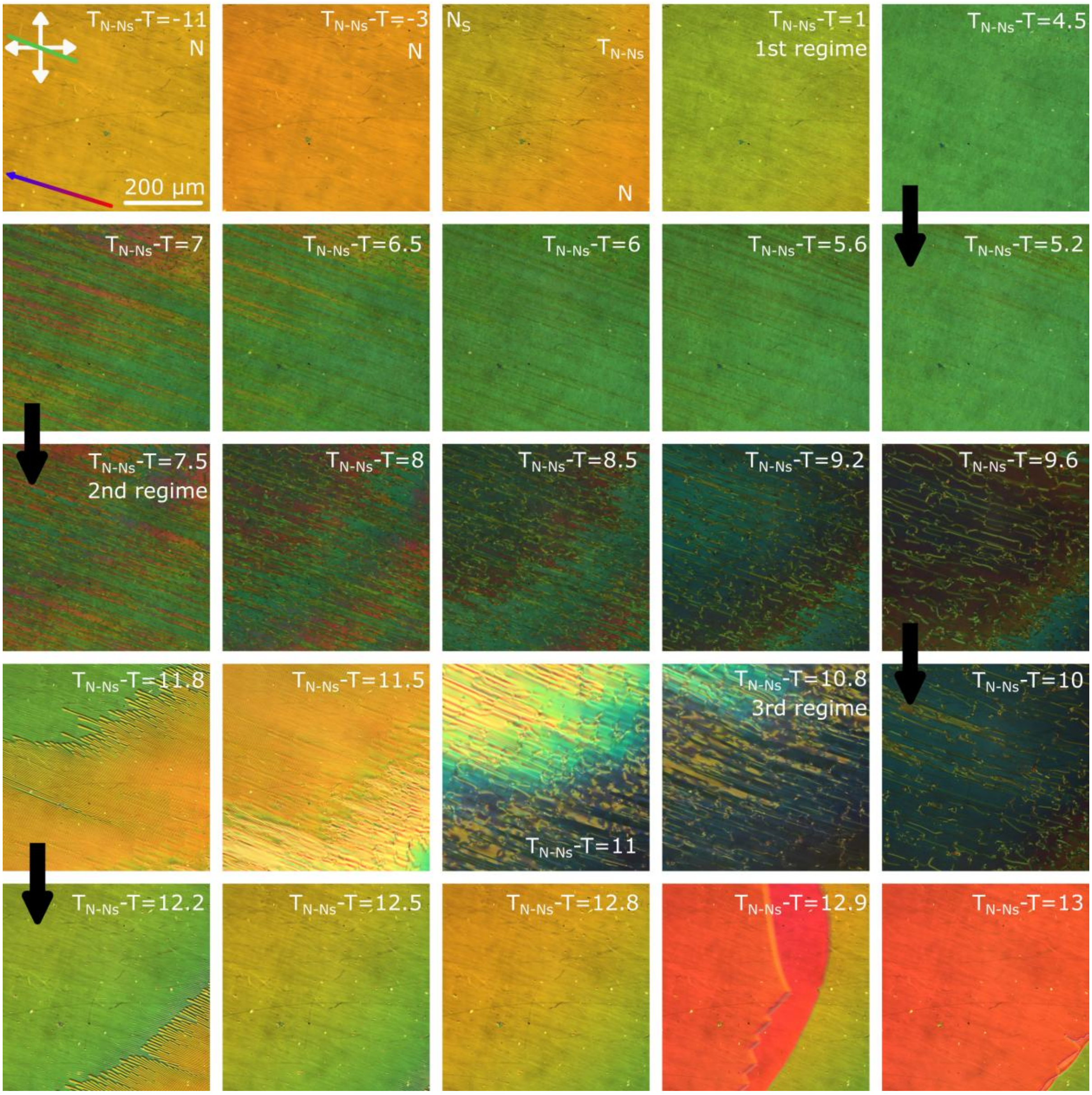

Figure S1. Polarizing optical microscopy images of the textures of RM734 + 0.5 wt% [BMIM][PF_6] in a 5μm thick cell with parallel rubbing under a small temperature gradient during a cooling run. Double-headed arrows indicate the direction of the crossed polarizers. The green line indicates the LC cell's rubbing direction. Gradient colored arrow indicates the direction of the temperature gradient, with the blue color (arrowhead) indicating the lower temperature end.

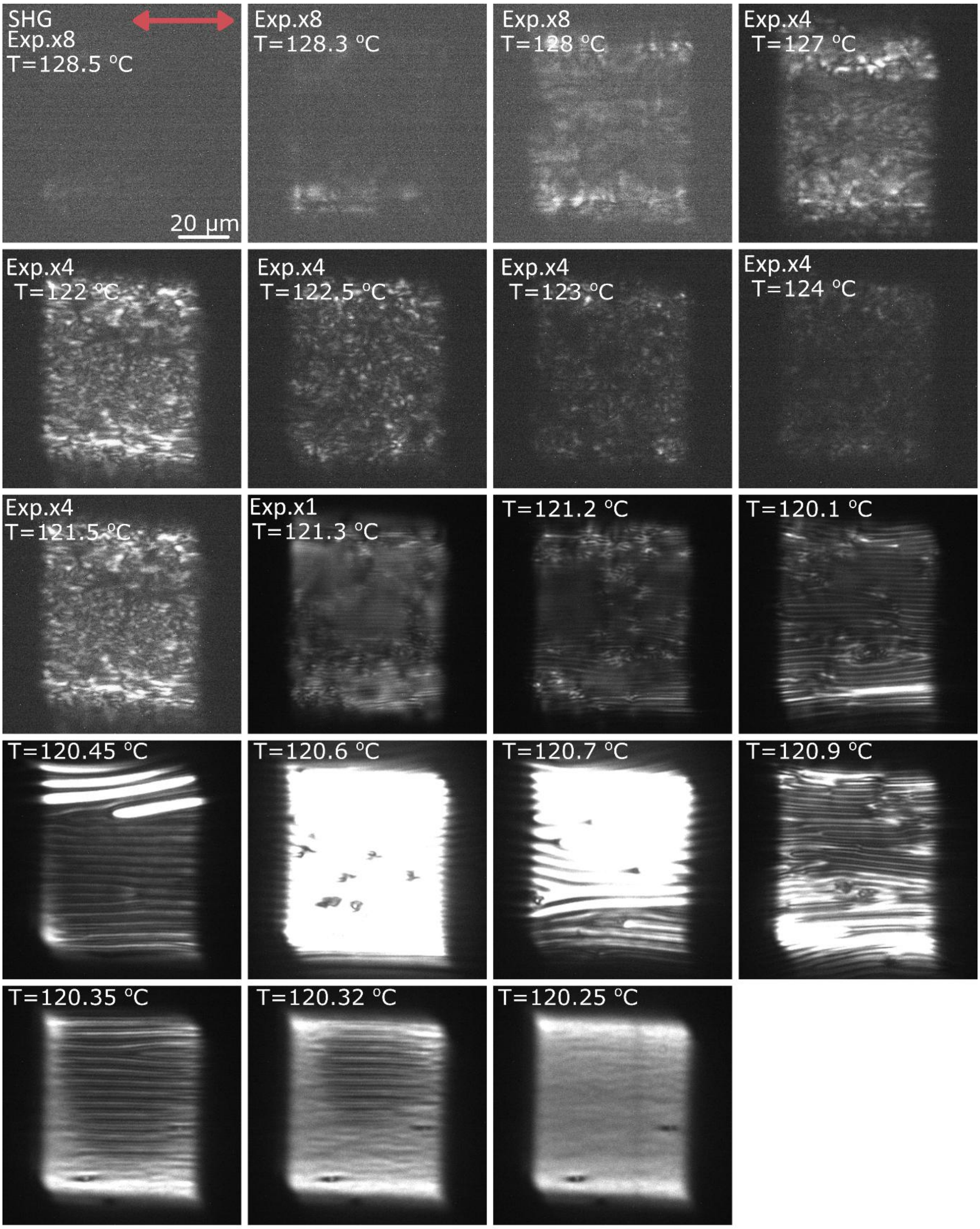

Figure S2. Second Harmonic Generation Microscopy (SHG-M) images of RM734+0.5 wt% [BMIM][PF_6] in a 5μm thick cell with parallel rubbing during a cooling run. Double-headed arrow indicates the direction of the incoming light polarization. Images were taken without analyzer. The rubbing direction of the cell is horizontal in the images.

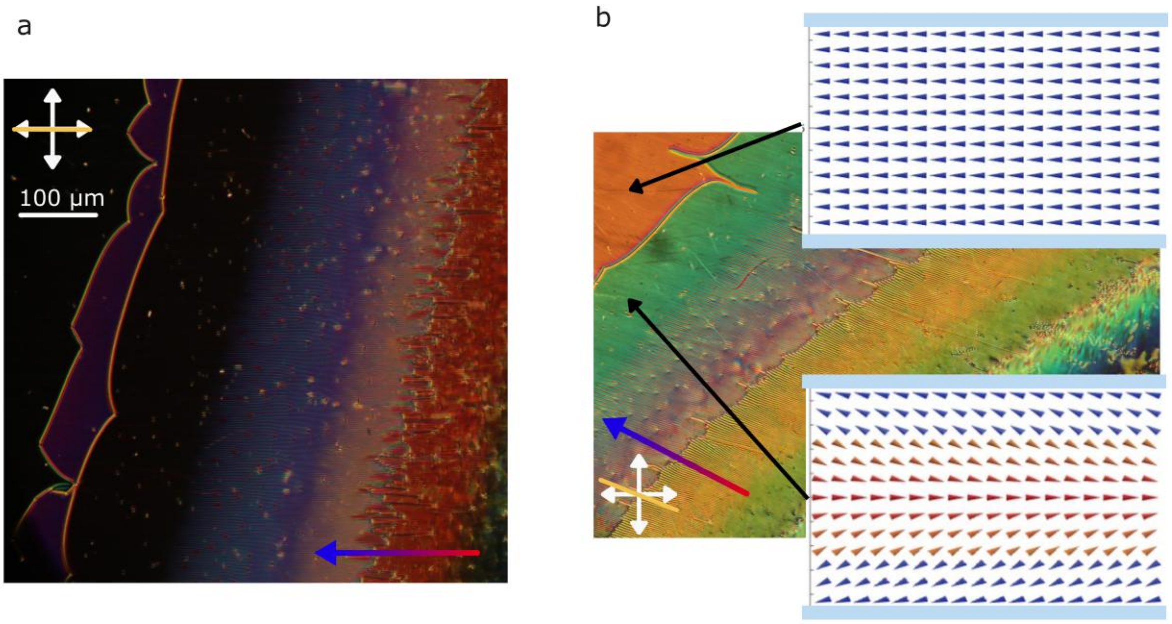

Figure S3. Polarizing optical microscopy images of the textures of RM734+0.5 wt% [BMIM][PF_6]. (a) At 110 °C, in a 5 μm thick cell with parallel rubbing highlighting the twisted structure that emerges between the two defect lines dividing homogeneous regions when not located together. (b) Such lines divide two homogeneous regions. This reveals different effective birefringence when the sample is rotated between crossed polarizers. This can be explained by the partial out-of-plane orientation of **n** due to a splay structure, as depicted in the image. Light blue rectangles represent the LC cell surfaces. The estimated difference in effective birefringence between both structures is 0.04, with lower temperature structure having larger value.

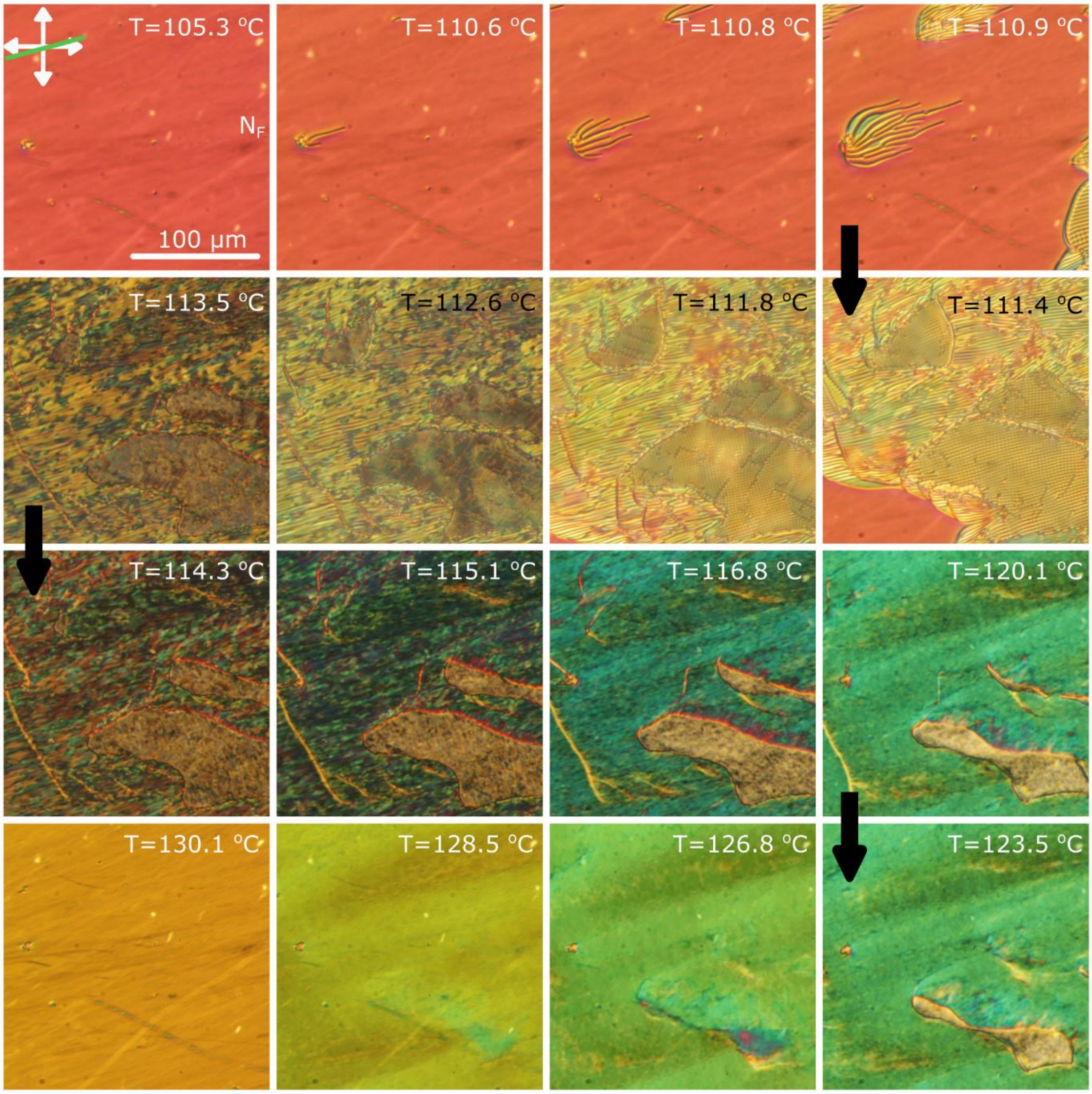

Figure S4. Polarizing optical microscopy images of the textures of RM734+0.5 wt% [BMIM][PF_6] in a 10 μm thick cell with parallel rubbing in a heating run (1 °C/min). Double-headed arrows indicate the direction of the crossed polarizers. The green line indicates the LC cell's rubbing direction.

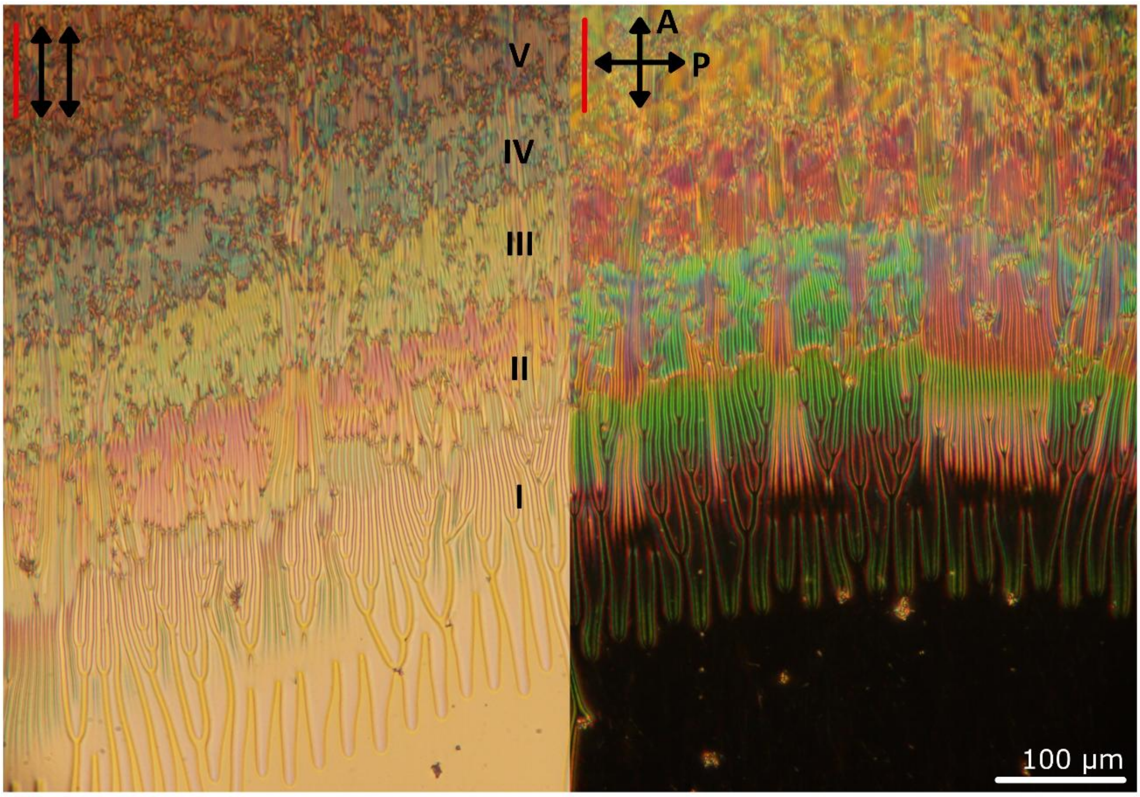

Figure S5. Polarizing optical microscopy images of the textures of RM734 + 0.5 wt% [BMIM][PF_6] in a 10 µm thick cell with parallel rubbing at 111 °C. Image is composed of two microphotograph between polarizers at different orientations, indicated by the double-headed arrows; parallel and parallel to the rubbing in the left and crossed in the right. The red line indicates the LC cell's rubbing direction.

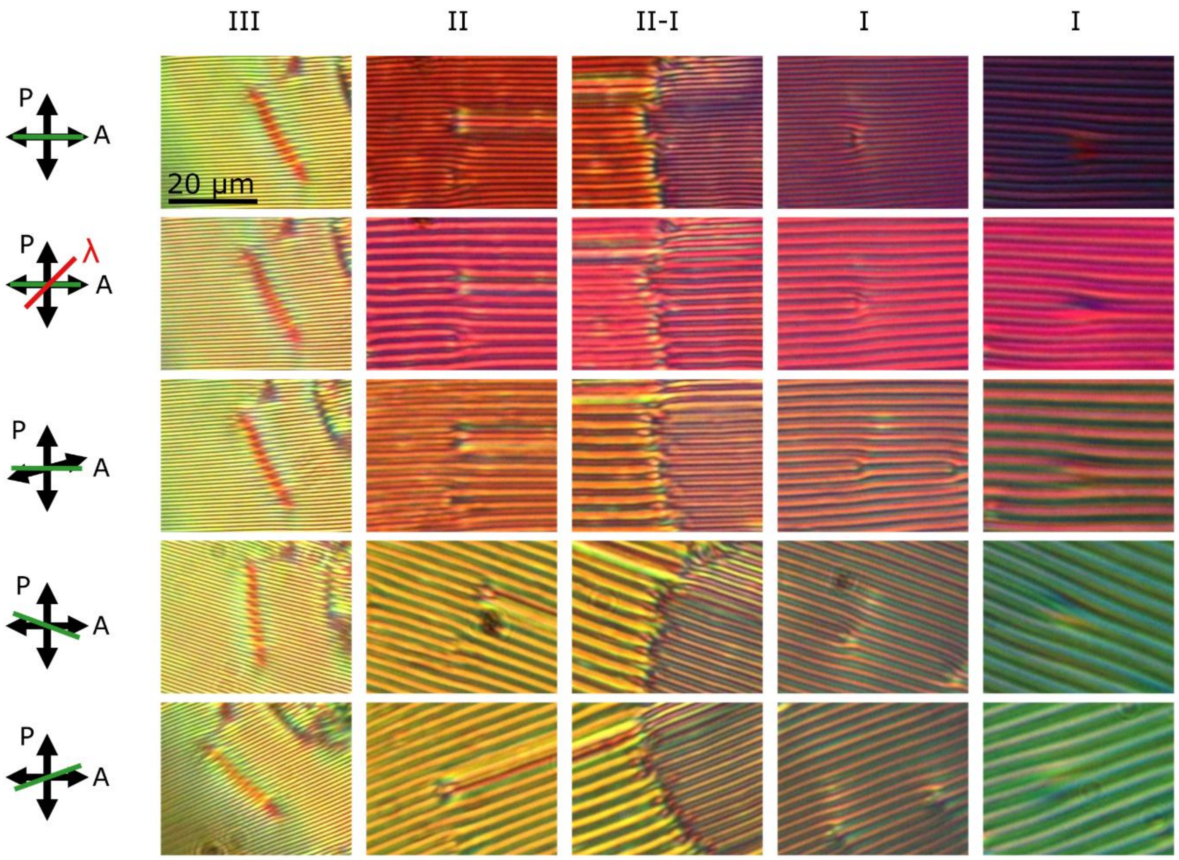

Figure S6. Zoom-in images from the highlighted areas in Figure 3 in the main manuscript at different conditions for for RM734 + 0.5 wt% [BMIM][PF_6] in 5 μm parallel rubbed cell. From top to bottom: under crossed polarizers with the cell rubbing direction aligned with the anal, with a full-wave plate inserted at 45 degrees, with analyzer uncrossed by 20 degrees, and with the sample rotated clock- and anticlockwise. Each column corresponds to the different discerned periodic structures, where **I** corresponds to the last one before homogenization.

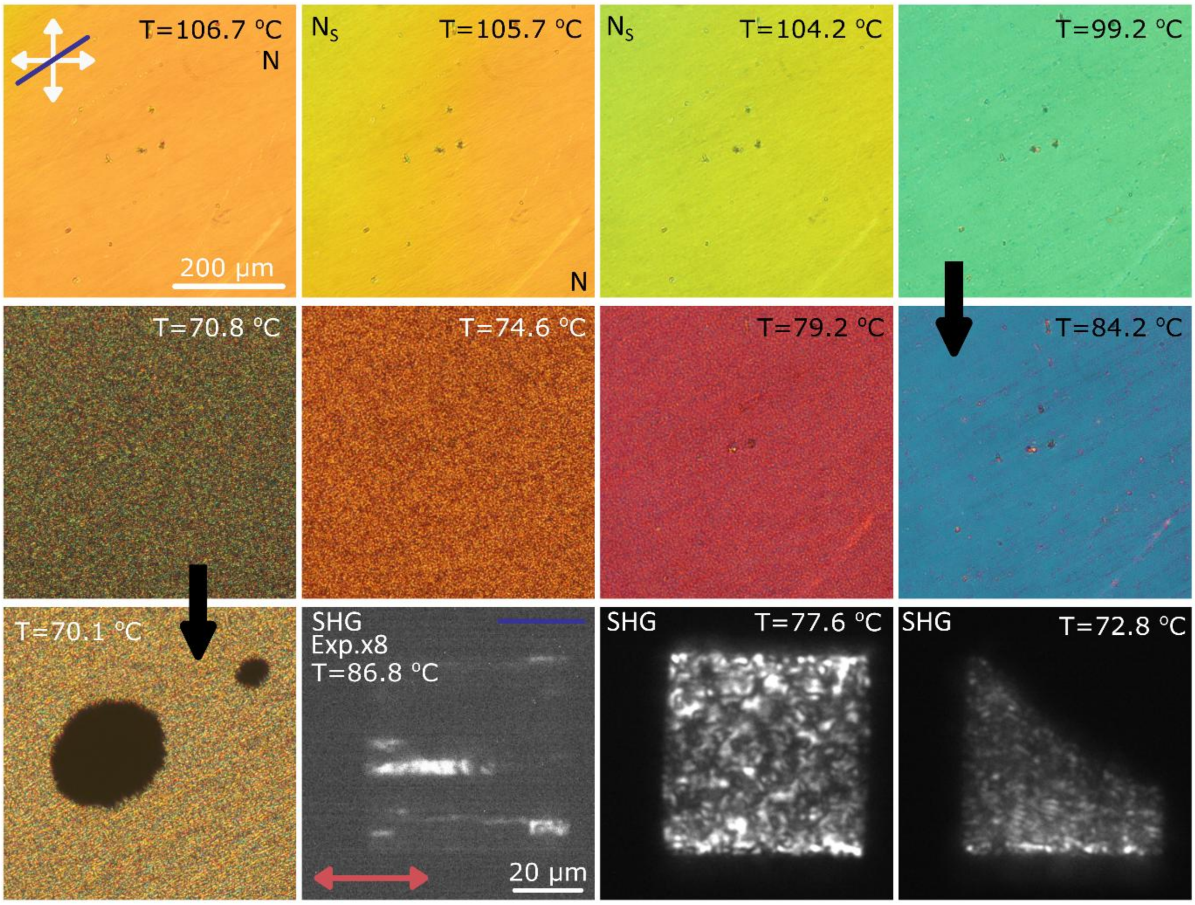

Figure S7. Polarizing optical microscopy images of the textures of RM734 + 5 wt% [BMIM][PF_6] in a 5 μm thick cell under cooling at 1 °C/min. 35 K below N-Ns transition the sample exhibits transition to a phase that is optically isotropic. Double-headed arrows indicate the direction of the crossed polarizers. The blue line indicates the LC cell's rubbing direction. In the bottom row, the last three images correspond to SHG-M observations at different temperatures. The sample becomes weakly visible in SHG-M at the N-Ns phase transition and remains so until a grainy structure appears. There the intensity of the M-SHG images becomes stronger and with further cooling decreases. When the material enters the optically isotropic phase, it completely disappears.

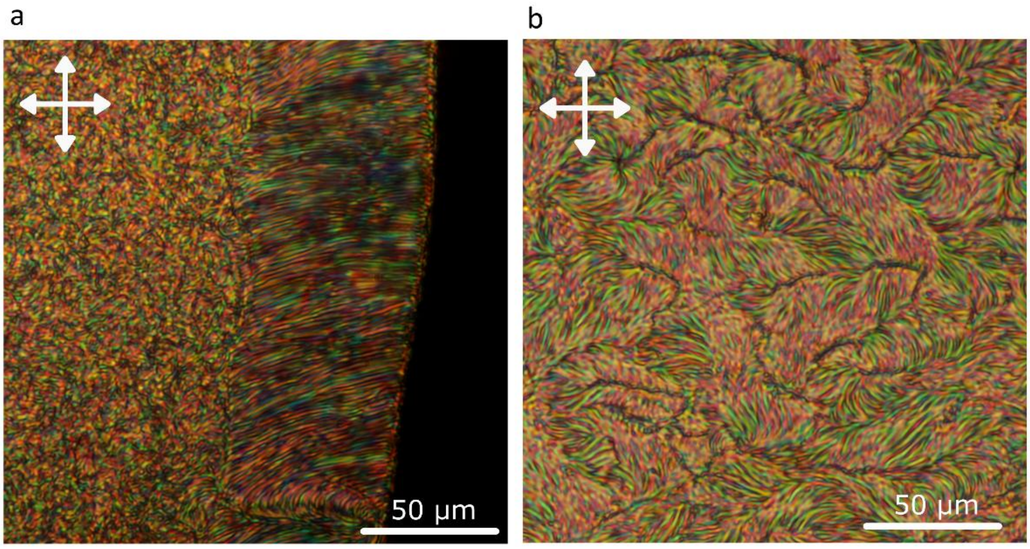

Figure S8. Polarizing optical microscopy images of the textures of FNLC-1571 + 0.6 wt% [BMIM][PF_6] in a 10 µm cell with surfaces coated with nonafluorohexyltriethoxysilane. Double-headed arrows indicated the direction of the crossed polarizers.

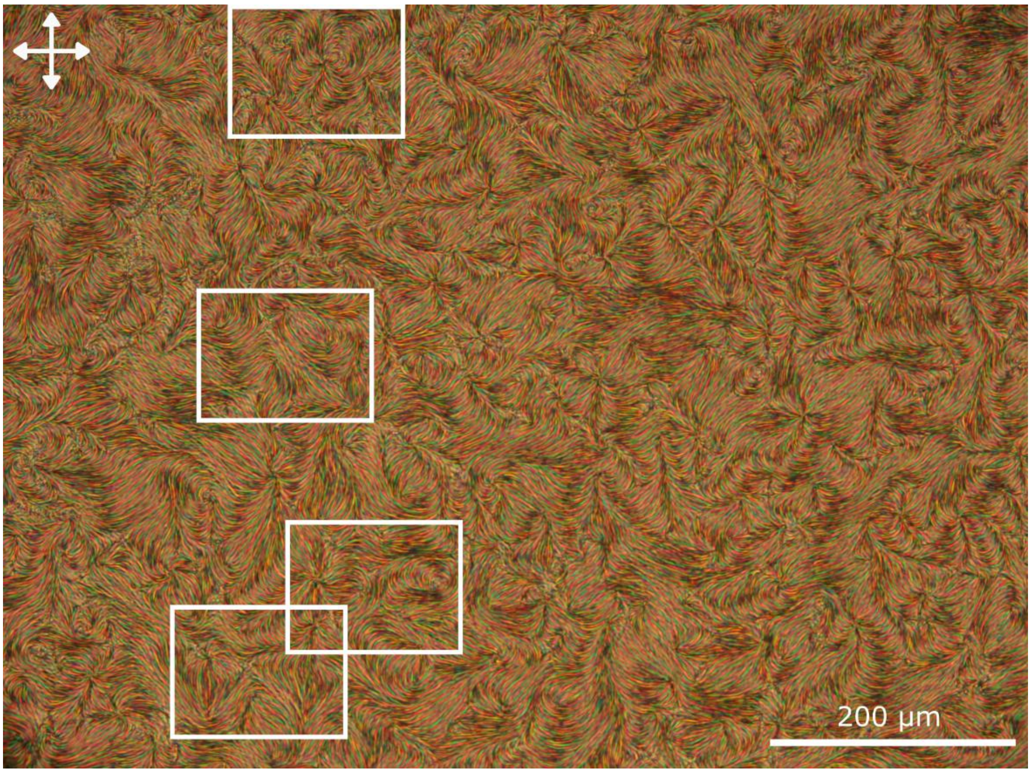

Figure S9. Polarizing optical microscopy textures observed for FNLC-1571 + 0.6wt% of [BMIM][PF_6] in a 10 µm LC cell without aligning layers at room temperature outside the ITO electrode area. Double-headed arrows indicate the direction of the crossed polarizers. Rectangles highlight the areas shown in Figure 6 in the main manuscript.

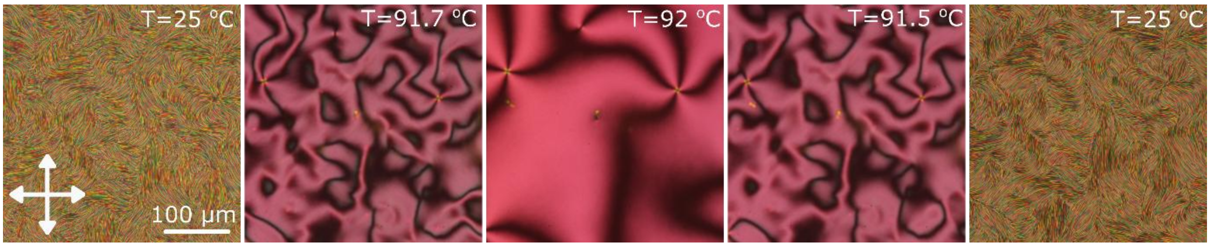

Figure S10. Polarizing optical microscopy textures observed for FNLC-1571 + 0.6wt% of [BMIM][PF_6] in a 10 μm LC cell without aligning layers outside the ITO electrode area upon heating from room temperature and subsequent cooling at 0.5 °C/min. Double-headed arrows indicate the direction of the crossed polarizers.

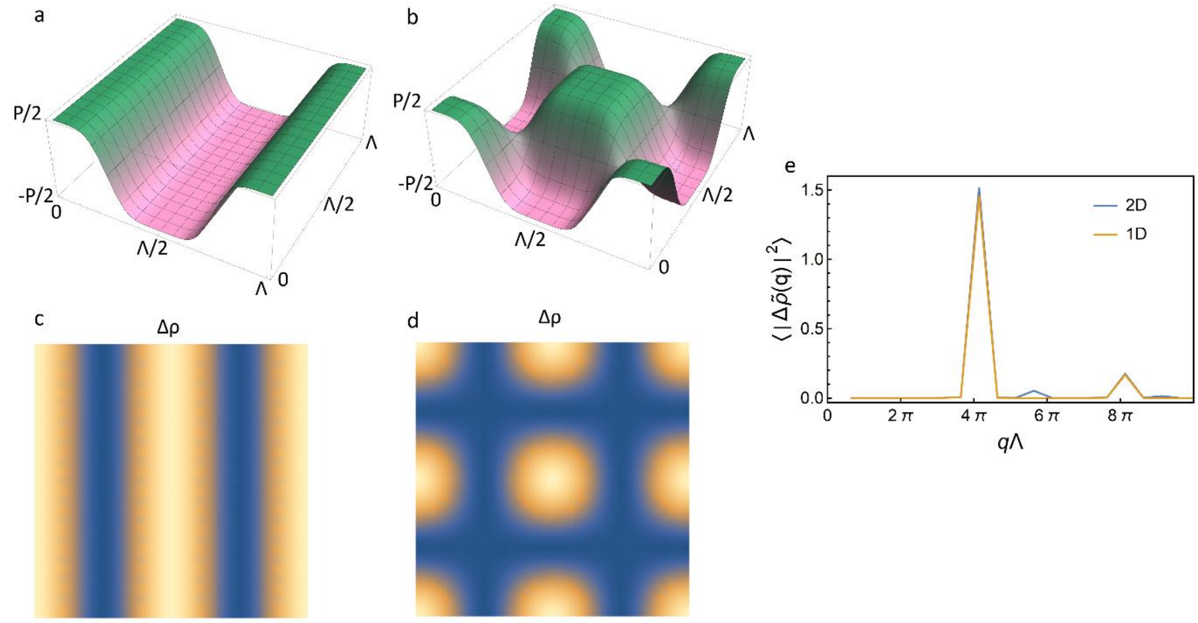

Figure S11. (a-b) 1D and 2D modulation in the polarization magnitude *P*. (c-d) Corresponding modulation in density, proportional to P^2, considering that the ferroelectric ordering increases the density of the material [1,2]. (e) Square of the Fourier transform of the density $\langle|\Delta\tilde{\rho}(q)|^2\rangle$ for the structures in (a-d).

Supplementary Note 1

Similarly as it was done in the description of the patterned structures in the N$_F$ phase [34], the electrostatic terms were added to the free energy density used for the description of the pretransitional behavior at the N-N$_S$ transition [3,4].

In this model, the following assumptions are made: (i) S is constant, so the nematic order can be described only by $\mathbf{n}$; (ii) $\mathbf{P} = \mathbf{P}_s + \boldsymbol{\varepsilon}_0(\boldsymbol{\varepsilon} - \mathbf{I})\mathbf{E}$, where $\mathbf{P}_s = P(x)\mathbf{n}$; (iii) $\mathbf{n}$ lays in the *xz* plane $\mathbf{n} = \left(n_x(x), 0, \sqrt{1 - n_x(x)^2}\right)$, and (iv) the dielectric tensor is isotropic, $\boldsymbol{\varepsilon} = \varepsilon\mathbf{I}$. The relevant terms in the free energy can then be written:

$$F = \int \left(\tfrac{1}{2}K_1(\mathbf{n}\nabla \cdot \mathbf{n} - \mathbf{S}_0)^2 + \tfrac{1}{2}K_3|\mathbf{n} \times (\boldsymbol{\nabla} \times \mathbf{n})|^2 + \tfrac{1}{4}B(P^2 - P_0^2)^2 + \frac{1}{2}K_P(\nabla P)^2 + \tfrac{1}{2}(\rho_f - \nabla \cdot \mathbf{P})\phi\right) dV \qquad (1)$$

Here, K_i (i = 1,3) are the splay and bend elastic constants and γ is the flexoelectric coefficient. The flexoelectric term $-\gamma(\mathbf{n} \cdot \mathbf{P})\nabla \cdot \mathbf{n}$ is included in the first term, where $\mathbf{S}_0 = \gamma\mathbf{P}/K_1$ is the ideal splay curvature, which would minimize the splay elastic energy. The sign of $\mathbf{S}_0$ determines the preferred direction of **P** when splay deformation is present in the system. The ideal splay curvature which would minimize the splay elastic energy, is $S_0 = \mathbf{n} \cdot \mathbf{S}_0 = \gamma P/K_1$. The first term is larger than the sum of the splay elastic and flexoelectric terms by $\tfrac{1}{2}K_1|\mathbf{S}_0|^2$, which is subtracted in the third term. The latter combines the Landau quadratic and quartic terms $\frac{1}{2}AP^2 + \frac{1}{4}BP^4$, with A and B being the corresponding coefficients and $P_0^2 = \left(\frac{\gamma^2}{K_1} - A\right)/B$. The fourth term is the first term allowed by symmetry in ∇P, and the last term is the electrostatic term with $\rho_f = \rho^+ - \rho^-$, and ϕ the electrostatic potential. Assuming that the free charge density follows the Boltzmann distribution $\rho^\pm = \pm\rho_0 \mathrm{Exp}\left(\mp e\Phi/(k_B T)\right)$, and that positive and negative ions carry a charge $e = \pm Z e_0$, (where e_0 is the elementary charge, Z positive integer, and the ion density ρ_0 is the same for positive and negative free ions), then $\rho_f = -2\rho_0 \sinh\left(e\Phi/(k_B T)\right)$. If $|e\Phi/(k_B T)| < 1$, then $\rho_f = -\frac{2e\rho_0}{k_B T}\phi$, and ϕ can be calculated using the linearized Poisson-Boltzmann equation $\nabla^2\phi = \kappa_D^2\phi + \frac{1}{\varepsilon\varepsilon_0}\nabla \cdot \mathbf{P}$, where $\kappa_D^{-1}$is the Debye screening length $\kappa_D^{-1} = \sqrt{\frac{\varepsilon\varepsilon_0 k_B T}{2\, e\rho_0}}$. The bound charges are given by $\rho_b = -\nabla \cdot \mathbf{P}$. We further assumed that the modulated splay structure consists of domains with a constant magnitude of polarization P_{max} and a constant splay deformation, i.e., constant splay curvature $S = s_d S_0$, with neighboring domains having opposite signs of polarization. The domains are separated by Ising domain walls with thickness d_w, in which polarization magnitude linearly changes from + P_{max} to – P_{max} or vice versa, and $n_x(x) = \pm(n_{x0} - k(x - x_{wc})^2)$, where x_{wc} is the position of the domain center, n_{x0} the splay amplitude and the coefficient k depends on the Λ, d_w, and s_d. It is also assumed that the sample is much larger than the Debye length so that the surface of the sample doesn't affect the structure in the bulk.

For such a structure, the electrostatic potential can be analytically calculated. Using the units of κ_D^{-1} for x, the solution for normalized electrostatic potential $\phi_n = \frac{e\phi}{k_B T}$ in the domain is

$$\phi_{n,d}[x] = -\frac{4\,\pi\xi_B}{\kappa_D^2}\frac{P_{max}S_0 s_d}{{d_w}^2}(1 + C_d[d_w, \Lambda]\cosh(x - x_{dc})) = -\frac{P_{max}S_0 s_d}{{d_w}^2}h_{d,es}[\Lambda, d_w, x] \qquad (2)$$

where $C_d[d_w, \Lambda] = \left((24 - \Lambda\, d_w + 2\, d_w^2)\sinh\left(\frac{d_w}{2}\right) - 12\, d_w \cosh\left(\frac{d_w}{2}\right)\right) / \left(2\sinh\left(\frac{\Lambda}{4}\right)\right)$, x_{dc} the position of the domain center, and $\xi_B = \frac{e_0^2}{4\pi\varepsilon\varepsilon_0 k_B T}$ Bjerrum length.

The solution for the domain wall reads:

$$\phi_{n,w}[x] = -\frac{4\pi\xi_B}{\kappa_D^2}\frac{P_{max}S_0 s_d}{{d_w}^2}\left(12 - \frac{\Lambda d_w}{2} + \frac{d_w^2}{2} + 6(x - x_{wc})^2 + C_d[d_w, \Lambda]\cosh(x - x_{wc})\right) = -\frac{P_{max}S_0 s_d}{{d_w}^2} h_{w,es}[\Lambda, d_w, x] \tag{3}$$

where $C_w[d_w, \Lambda] = \left((-24 + \Lambda\, d_w - 2\, d_w^2)\sinh\left(\frac{\Lambda - 2d_w}{4}\right) - 12\, d_w \cosh\left(\frac{\Lambda - 2d_w}{4}\right)\right) / \left(2\sinh\left(\frac{\Lambda}{4}\right)\right)$, and x_{wc} the position of the domain wall center.

The free energy (Eq. 1) is then a function of Λ, s_d, d_w, and P_{max}. In the domains,

$$F_D = A_{yz}\int_{-(\Lambda - 2d_w)/2}^{(\Lambda - 2d_w)/2}\left(\frac{1}{2}K_1 S_0^2 (s_d - 1)^2 + \frac{1}{2}K_3 S_0^4 s_d^4 \frac{(x - x_{dc})^2}{1 - S_0^2 s_d^2 (x - x_{dc})^2} + \frac{1}{4}B(P_{max}^2 - P_0^2)^2 + f_{d,es}\right)dx \tag{4}$$

and in the walls:

$$F_w/A_{yz} = \int_{-d_w/2}^{d_w/2}\left(2K_1 S_0^2 (s_d - 1)^2 \frac{(x - x_{dc})^2}{d_w^2} + 2K_3 S_0^4 s_d^4 \frac{h_B[x - x_{wc}]}{d_w^2} + \frac{1}{4}B\left(P_{max}^2 \frac{4(x - x_{wc})^2}{d_w^2} - P_0^2\right)^2 + \frac{1}{2}K_P\left(\frac{2P_{max}}{d_w}\right)^2 + f_{w,es}\right)dx \tag{5}$$

where

$$f_{d,es} = \frac{{P_{max}}^2 S_0^2 s_d^2}{{d_w}^4}\left(-2en_0\, h_{d,es}[\Lambda, d_w, x - x_{dc}]^2 + \frac{k_B T}{2e}{d_w}^2 h_{d,es}[\Lambda, d_w, x - x_{dc}]\right),$$

$$f_{w,es} = \frac{{P_{max}}^2 S_0^2 s_d^2}{{d_w}^4}\left(-2en_0\, h_{w,es}[\Lambda, d_w, x - x_{wc}]^2 + \frac{k_B T}{4e}(12(x - x_{w,c})^2 - d_w(\Lambda - \mathrm{d_w}))h_{w,es}[\Lambda, d_w, x - x_{wc}]\right),$$

$$h_B[x] = \frac{(4x^3 + x d_w(-\Lambda + d_w))^2}{(16 d_w^2 - S_0^2 s_d^2 (4x^2 + d_w(-\Lambda + d_w))^2)},$$

and A_{yz} is the *yz* surface of the sample.

The constraint for the splay amplitude, $n_{x0} \leq 1$, leads to the constrain for the modulation period $\Lambda \leq \frac{4}{S_0 s_d} + d_w$.

Typically, in the realistic cases, the 3rd term is larger than elastic and flexoelectric terms, and, consequently, $P_{max} \approx P_0$. This term favours small d_w. The first term is minimized for $s_d = 1$. The second, the bend elastic term becomes relevant when $S_0^2 s_d^2 (x - x_{dc})^2$ approaches 1, which is when n_{x0} is approaching 1. So the main effect of this term is suppressing the splay amplitude. The fifth, ie., the polarization gradient term differs from 0 only in the domain wall, and favours large d_w. The electrostatic term favours small s_d and large d_w.

The numerical minimization of the free energy were performed for the following set of parameters $K_1 = K_3 = 20$ pN, $\gamma = -0.01$ V, $A = \frac{A_n}{(\varepsilon\varepsilon_0)}$, $B = 4.5 \cdot 10^9 \frac{\mathrm{Nm}^6}{(\mathrm{As})^4}$, $K_P = 8 \cdot 10^{-10}\ \mathrm{Nm}^4/(\mathrm{As})^2$, $\varepsilon = 100$, and $Z = 1$. Here, the parameter A_n plays the role of the temperature, and parameter *B* is chosen

so that at $A_n = 1$, $P_0 = 0.05\ \mathrm{As/m^2}$. The integrand in Eq. 1 was numerically averaged over one modulation period for discrete sets of allowed values of Λ, s_d, and d_w while $P_{max} = P_0$ was fixed. The set with the minimal averaged value of the free energy was chosen as a solution for Λ, s_d, and d_w . The minimal averged value was then compared with the free energy density of uniform N_F phase ($n_{x0} = 0$, $P_0 = \sqrt{\frac{-A}{B}}$) to assess the stability of the N_S phase.

Note: When approaching the threshold value of $A_{n,tr}$ at which the transition to N_F happens, the value of the normalized electrostatic potential in the domain walls ϕ_n exceeded 1 which means that the linearization of Poisson Boltzmann equation is not that accurate anymore. Comparison of the solution of the linearized Poisson Boltzmann equation with the numerical solution of the full Poisson Boltzmann equation showed that the linearization overestimates ϕ_n in the domain walls for up to 20% when approaching $A_{n,tr}$. Consequently, the electrostatic energy is also over estimated, which leads to lower $A_{n,tr}$ and smaller Λ compared to those given by solutions of the linearized equation.

References

[1] C. Parton-Barr, H. F. Gleeson, and R. J. Mandle, “Room-temperature ferroelectric nematic liquid crystal showing a large and diverging density,” Soft Matter, **20**, 672 (2024).

[2] R. J. Mandle, N. Sebastián, J. Martinez-Perdiguero, and A. Mertelj, “On the molecular origins of the ferroelectric splay nematic phase,” Nat Commun, **12**, 4962 (2021).

[3] A. Mertelj, L. Cmok, N. Sebastián, R. J. Mandle, R. R. Parker, A. C. Whitwood, J. W. Goodby, and M. Čopič, “Splay Nematic Phase,” Physical Review X, **8**, 041025 (2018).

[4] N. Sebastián, L. Cmok, R. J. Mandle, M. R. de la Fuente, I. Drevenšek Olenik, M. Čopič, and A. Mertelj, “Ferroelectric-Ferroelastic Phase Transition in a Nematic Liquid Crystal,” Phys. Rev. Lett., **124**, 037801 (2020).
